# Functional and Physical Outcomes following Use of a Flexible CO_2_ Laser Fiber and Bipolar Electrocautery in Close Proximity to the Rat Sciatic Nerve with Correlation to an In Vitro Thermal Profile Model

**DOI:** 10.1155/2015/280254

**Published:** 2015-01-28

**Authors:** A. M. Robinson, A. J. Fishman, B. R. Bendok, C.-P. Richter

**Affiliations:** ^1^Department of Otolaryngology Head and Neck Surgery, Northwestern University, 303 E. Chicago Avenue, Searle 12-561, Chicago, IL 60611, USA; ^2^Cadence Neuroscience Institute at Northwestern Medicine, 25 N. Winfield Road, Winfield, IL 60190, USA; ^3^Department of Neurological Surgery, Northwestern University, 676 North St. Clair, Chicago, IL 60611, USA; ^4^Department of Biomedical Engineering, Northwestern University, 2145 Sheridan Road, Tech E310, Evanston, IL 60208, USA; ^5^The Hugh Knowles Center, Department of Communication Sciences and Disorders, Northwestern University, 2240 Campus Drive, Evanston, IL 60208, USA; ^6^Department of Otolaryngology Head and Neck Surgery, Feinberg School of Medicine, Northwestern University, Searle Building 12-470, 303 E. Chicago Avenue, Chicago, IL 60611-3008, USA

## Abstract

This study compared functional and physical collateral damage to a nerve when operating a Codman MALIS Bipolar Electrosurgical System CMC-III or a CO_2_ laser coupled to a laser, with correlation to an in vitro model of heating profiles created by the devices in thermochromic ink agarose. Functional damage of the rat sciatic nerve after operating the MALIS or CO_2_ laser at various power settings and proximities to the nerve was measured by electrically evoked nerve action potentials, and histology of the nerve was used to assess physical damage. Thermochromic ink dissolved in agarose was used to model the spatial and temporal profile of the collateral heating zone of the electrosurgical system and the laser ablation cone. We found that this laser can be operated at 2 W directly above the nerve with minimal damage, while power settings of 5 W and 10 W resulted in acute functional and physical nerve damage, correlating with the maximal heating cone in the thermochromic ink model. MALIS settings up to 40 (11 W) did not result in major functional or physical nerve damage until the nerve was between the forceps tips, correlating with the hottest zone, localized discretely between the tips.

## 1. Introduction

The first electrocautery device to be used in an operating room was essentially a spark generator, which never became popular [1]. A similar but revolutionary device (termed the Bovie device) also permitted cutting and was the basis of neural electrocautery for around fifty years [2]. It utilized a large ground plate and a cautery probe and became known as a unipolar type system when Greenwood introduced two pronged bipolar forceps with insulated prongs across which a current would pass [3]. Independently, Malis developed a similar laboratory tool for use on monkeys and in the absence of federal medical device regulations had a device made for the operating room [4]. He quickly made improvements to extend the life of the prongs and to maintain their separation distance over usage and had a new version of the device manufactured [5, 6]. Cutting limitations were improved with tungsten forceps, and the Codman MALIS Bipolar Electrosurgical System CMC-III came into surgical use [7].

The invention of the CO_2_ laser in 1963 proved to be particularly important for surgical procedures [8–11]. Carbon dioxide lasers emit infrared radiation around 10 *μ*m. Water in tissues efficiently absorbs infrared photons at this wavelength and converts their energy into heat. Laser tissue interaction is determined by the tissue properties. Tissue properties include the absorption coefficient and heat transfer. The combination of laser settings and tissue properties determines the rate of tissue heating, the area of laser effect, the amount of tissue ablated (cutting), coagulation, and thermal and collateral tissue damage, which may or may not be permanent [12].

For the laser as a surgical tool, ablation with no collateral tissue damage and control of bleeding are desired. Early CO_2_ laser systems had an open beam path which required cumbersome apparatus to direct the beam, which made handling in a clinical setting difficult [13–15]. With the invention of waveguides (OmniGuide, Inc., Cambridge, MA) infrared radiation from the laser can be directed with a thin hollow optical fiber and a handpiece, directly to the surgical site. The use of the CO_2_ laser in small confined areas and for microsurgical purposes can be performed [16–20].

Knowledge of the benefits and limitations of using lasers versus electrical surgical systems such as the MALIS Bipolar Electrosurgical System is important to optimize surgical procedures. Both lasers and bipolar electrocautery devices damage tissues by absorption of heat. Surgical lasers achieve this by delivery of radiation in the infrared wavelength of the electromagnetic spectrum. The purpose of this study was to determine the safe minimal proximity that can be made to minimize collateral damage to critical structures such as the sciatic nerve, while using either the laser or the electrosurgical system.

Stimulation and measurement of electrode potentials of the sciatic nerve provided acute measures of nerve function in response to proximal surgery in progress. Loss of nerve function and physical damage were correlated to heating profiles created by the surgical devices in an in vitro thermochromic ink agarose artificial tissue model.

## 2. Methods

### 2.1. Ethics Statement

All animal procedures were carried out within the guidelines of the NIH Guide for the Care and Use of Laboratory Animals and were approved by the Animal Care and Use Committee of Northwestern University.

### 2.2. Animal Surgery

Rats of either sex (200–300 g) were anesthetized by an initial intraperitoneal (i.p.) injection of ketamine (60–90 mg/kg) and xylazine (6–9 mg/kg) in the lower abdomen. For maintenance of anesthesia, 20–33% of the original dose of ketamine/xylazine was given if the animal showed signs of increasing arousal, which was tested with a paw withdraw reflex, or if the heart or the respiratory rate increased. Body temperature was monitored and maintained at 38°C with a rectal probe and a heating blanket (Homeothermic Blanket Control Unit, Harvard Apparatus, Holliston, MA). After the animal was anesthetized, it was placed belly up on the heating blanket. The sciatic nerve was surgically exposed and electrodes were put in place. The nerve and surrounding tissue were kept moist throughout the experiment by application of Ringer's lactate with suction of excess fluid as necessary. To ensure efficient use of animals an effort was made to use both the left and right side sciatic nerves in individual animals. The left and right sciatic nerve treatments within an animal were chosen randomly when possible and were different.

#### 2.2.1. Measurement of Compound Nerve Potentials (CNPs)

**Figure 1:**
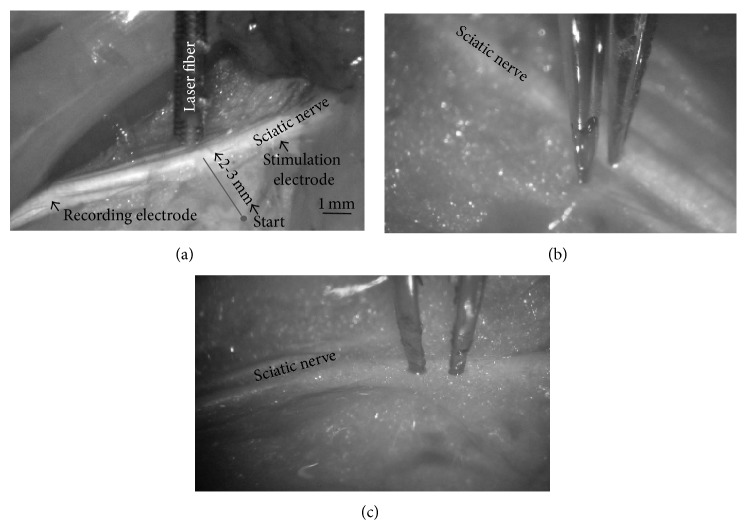
Photographs show the exposed sciatic nerves. The positions where two silver wires, 125 *μ*m in diameter, were placed in a small pocket below the nerve are indicated with arrows. The stimulating electrode is situated proximal to the body and was used to deliver biphasic electrical pulses (0.25 ms per phase) to evoke nerve compound action potentials (CNPs). The recording electrode was distal to the body and was used to monitor the CNPs. (a) shows the positioning of the OmniGuide Neuro-laser fiber. The distance from the nerve was controlled using a micromanipulator and the tip of the fiber was placed 2 mm above the tissue. The starting point for the experiment was 2-3 mm lateral from the gray line. (b) shows the MALIS forceps tines in contact with the tissue, perpendicular to the nerve and parallel to the nerve in (c).

To monitor sciatic nerve function, a stimulating electrode (125 *μ*m diameter silver wire) was placed proximally under the sciatic nerve and a return electrode was inserted into a pocket under the skin. Electrode placement positions are indicated in Figure 1(a). The electrodes were connected to a current source (Valhalla Scientific, Poway, CA), which was controlled with custom written software using TestPoint (SuperLogics Inc., Natick, MA). The software generated biphasic electrical pulses (0.25 ms per phase) that were delivered at current amplitudes between 0.3 and 0.8 mA to stimulate the sciatic nerve. An electrode (125 *μ*m diameter silver wire with a small ball at its end) served to record the nerve action potentials. It was placed about 8 mm distal to the stimulation electrode under the sciatic nerve, with the reference electrode inserted into a pocket below the skin. The leads were connected to the head stage of a DMA 50 amplifier (World Precision Instruments, Sarasota, FL). Nerve responses were amplified by 60 dB, were bandpass-filtered (0.1–3 kHz), were digitized with a KPCI 3110 *I*/*O* board (Keithley, Cleveland, OH), and were stored on a PC at a sampling rate of 100 kHz.

### 2.3. Use of Surgical Tools

#### 2.3.1. The CO_2_ Laser

The Neuro-laser fiber (OmniGuide Inc., Cambridge, MA) was coupled to the output of the OmniGuide CO_2_ laser system and was inserted into a handpiece, which was mounted to a MHW3-micromanipulator (Narishige International USA, Inc., East Meadow, NY). The laser fiber was set at 2 mm above the tissue and moved in perpendicular steps of 200 *μ*m towards the nerve from either 2000 or 3000 *μ*m away (Figure 1(a)). At each new position, a laser pulse (100 ms) was delivered and the nerve function was tested by measuring CNPs as described. CNP measurements were repeated for different laser power settings: 2, 5, and 10 W. The actual average laser power delivered at the fiber tip was measured in the continuous wave mode (100 ms pulse length and 10 Hz repetition rate) with a 3Sigma Coherent with a power sensor PM150-50C and was 0.9 ± 0.06, 2.3 ± 0.22, and 4.8 ± 0.03 W for the 2, 5, and 10 W power settings, respectively. The corresponding energies per pulse were 90 ± 6, 230 ± 20, and 480 ± 30 mJ.

#### 2.3.2. The Codman MALIS CMC-III Electrosurgical System

A Non-Stick Mirror Finish Bipolar Forceps (0.25 mm tip diameter, Codman, Johnson and Johnson) was placed with its prongs perpendicular (Figure 1(b)) or parallel (Figure 1(c)) to the sciatic nerve. The distance between the tips was fixed at 0.5 mm. The bipolar forceps was connected to a MALIS Bipolar Electrosurgical System (CMC-III, Codman, Johnson and Johnson). The forceps tips were initially placed 2000 or 3000 *μ*m lateral to the nerve and 0.5 mm deep into the tissue. The MALIS was activated for 2 s (this corresponds to the time to effect) in its cutting mode at either 30 MALIS (7 W) or 40 MALIS (11 W) setting. The tips were then retracted above the tissue and moved in steps of 250 *μ*m towards the nerve, lowered 0.5 mm into the tissue, and retracted upward following each activation. Collateral functional damage to the sciatic nerve was repeatedly monitored by measuring CNPs as described.

### 2.4. Histology

Sciatic nerve sections encompassing the microsurgical target sites were dissected and placed immediately into cold 4% paraformaldehyde in 0.14 M, pH 7.4 phosphate buffered saline (PBS). Following approximately 24 hours of fixation, the nerves were washed in PBS at room temperature for 2 hours. Nerves were dehydrated in graded ethanols, cleared in xylene, and embedded in paraffin wax. Longitudinal sections (7 *μ*m) of the nerve segments were cut and mounted on glass slides for histology. After staining with hematoxylin or the Luna stain [21], sections were dehydrated, cover slips were applied, and digital images were captured for histological evaluation.

### 2.5. In Vitro Thermochromic Ink Agarose Measurements

**Figure 2:**
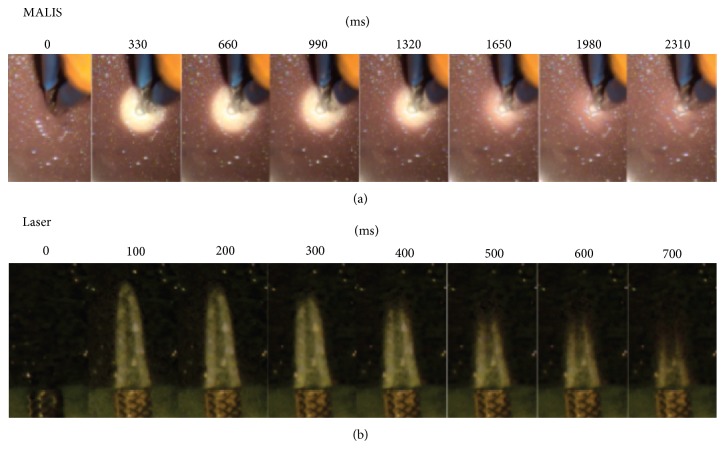
Temperature profiles of the MALIS and laser pulse following activation, using in vitro thermochromic ink gel model. The top row shows the increased temperature (white area) and thermal dissipation over time, given in milliseconds (ms) following 100 ms of MALIS activation. The bottom row shows the increased temperature (white cone) after delivery of a laser pulse. The heat dissipated within 750 *μ*m and the temperature increase extension beyond the ablation cone was less than 300 *μ*m.

Thermochromic ink (Chromatic Technologies Inc., Colorado Springs, CO) was used to image the spatial and temporal temperature changes following laser pulses or MALIS activation. The thermochromic ink is black below a specific temperature and becomes gray to colorless when the temperature exceeds the nominal temperature. The color change can be used to determine the temperature change. The ink was dissolved in 1% solution of agarose (Sigma-Aldrich, St. Louis, MO) to make a ~10% ink solution in a 3 mm thick agarose slab. Since the ink-agarose gel is primarily composed of water, infrared radiation (IR) from the laser is absorbed and causes an increase in temperature, which is documented by the change in color (black to gray/colorless) of the ink within the gel. The laser fiber was mounted to a 3D micromanipulator and oriented towards the ink slab so that the beam path was parallel to the surface of the slab and the MALIS tips were 0.5 mm into the agarose (Figure 2). For each MALIS activation or laser pulse the change in color was recorded via images captured over time with a digital camera (Sony, DCR-TRV) (Figure 2). To create a calibration curve of temperature with ink color the agarose slab was immersed in a 41°C distilled water bath, which was allowed to cool down, and images were captured at each 1°C drop in its temperature. The color changes were converted into temperature changes. A sigmoid function was plotted to the resulting data. The function was then used to convert ink color intensity values from the laser fiber measurements into temperature values. Subsequent sections were combined and a three-dimensional reconstruction of the temperature distribution was made.

### 2.6. Data Analysis

The amplitude from the first negative to the first positive peak of the evoked compound nerve action potentials (CNPs) was measured. Baseline CNPs were established at initial placement of the laser fiber and the bipolar forceps. The effect of distance from the nerve on CNP was determined for both. At least 5 CNPs (one per minute) were measured after each laser or forceps use at each new position (Figure 8). The CNP amplitudes at each new position were averaged and plotted versus the lateral distance from the nerve. Laser power settings were 2, 5, and 10 W; MALIS settings were 30 and 40 MALIS.

### 2.7. Statistics

Mean and standard deviations were calculated for compound nerve action potentials (CNPs) of the sciatic nerve for the different power settings and the varying lateral distances of the surgical system used (MALIS or laser) from the nerve. An analysis of variance (ANOVA) was performed. If the ANOVA indicated differences among the variances, an a posteriori test was used for making pairwise comparisons among the means. The honestly significant difference (HSD) test by Tukey was used. The tests are part of a statistical package provided by IGOR (Wavemetrics). Statistical decisions were made for a probability *P* = 0.05.

## 3. Results

### 3.1. Temperature Profiles versus Histology

#### 3.1.1. Temperature Profiles

Figure 2 shows the timeline of the local heating around the tips of the MALIS CMC-III bipolar forceps following activation for ~100 ms at 30 MALIS setting. A circular area of lightened ink indicates the region of temperature increase. The maximum diameter of the heated area is about 4 mm and the temperature decreases over a 2.3-second period after activation. In comparison, a 100 ms pulse from the CO_2_ laser (Figure 2) resulted in a confined heating cone of about 0.35 mm in width and about 1 mm in length. The cone has a hollow core indicating that the laser has ablated the agarose. The heat from the laser pulse dissipated over 700 ms.

**Figure 3:**
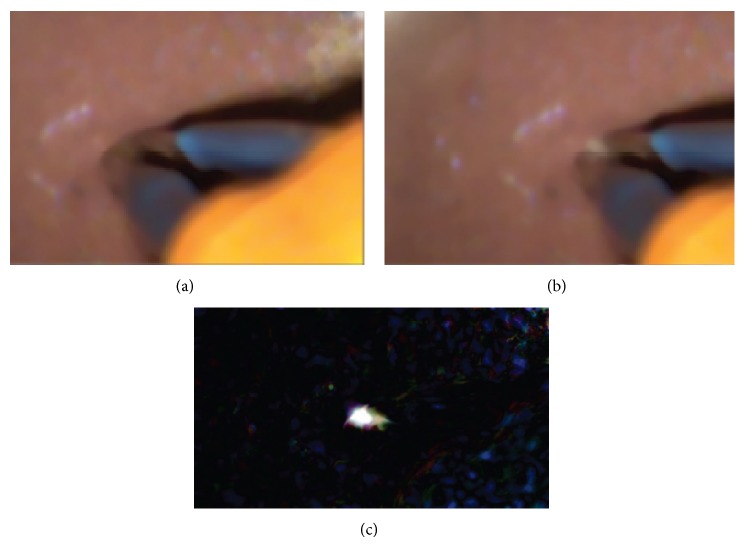
(a) shows the thermochromic ink gel between the MALIS forceps tips before activation and (b) during activation. (c) is the difference between images (a) and (b). The bright spot indicates the place of temperature increase, which is clearly localized between the forceps tips.

The MALIS activation time could be shortened until only the area between the tips showed a temperature change (Figure 3). Figures 3(a) and 3(b) show the tips of the forceps before and after activation, respectively. Subtraction of the two image pixel intensities (Figure 3(c)) shows that heating only occurs between the tips.

**Figure 4:**
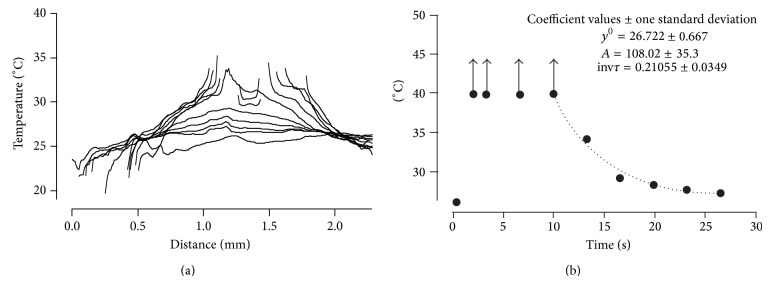
Upper plot shows the spatial temperature distribution for different time intervals after the laser pulse was delivered to thermochromic ink gel. The *x*-axis shows the distances from left to right across the diameter of the ablation cone. The *y*-axis is the temperature of the gel, each trace being a different time following the laser pulse delivery (upper traces are sooner after the pulse). The center of the ablation cone is at about 1.2 mm where the temperature was well above 35°C. It can be seen that immediately after the laser pulse for a diameter of about 1 mm the temperature increase is more than 10°C. Lower plot shows estimation of the maximum temperature in the center of the ablation cone by fitting the decrease in temperature over time with a simple exponential function. The maximum temperature was above 100°C.

The change in temperature of the agarose measured across the laser cone width over time after the laser pulse was plotted in Figure 4. The *x*-axis shows the distances from left to right across the cone diameter with the temperature on the *y*-axis. Each plotted line represents temperature measurements at different times following laser firing; uppermost plots are soonest after firing and therefore hottest. It can be seen that directly after the laser pulse for a diameter of about 1 mm the temperature increase is more than 10°C and the center of the ablation cone diameter is about 1.2 mm where the heating is suddenly much greater and above the limit of the ink calibration curve.

#### 3.1.2. Temporal Heat Distribution

The temporal resolution of the heat distribution was captured with a resolution of ~30 ms per frame. This makes it difficult to measure the temperature immediately at the delivery of the laser pulse. Since only one laser pulse was delivered, the distribution of the temperature was plotted over time (Figure 4(b)). Assuming that the temperature at a given location drops with a simple exponential function, the maximum temperature can be estimated and reaches well above 100°C at the center of the ablation cone diameter.

#### 3.1.3. Histology

**Figure 5:**
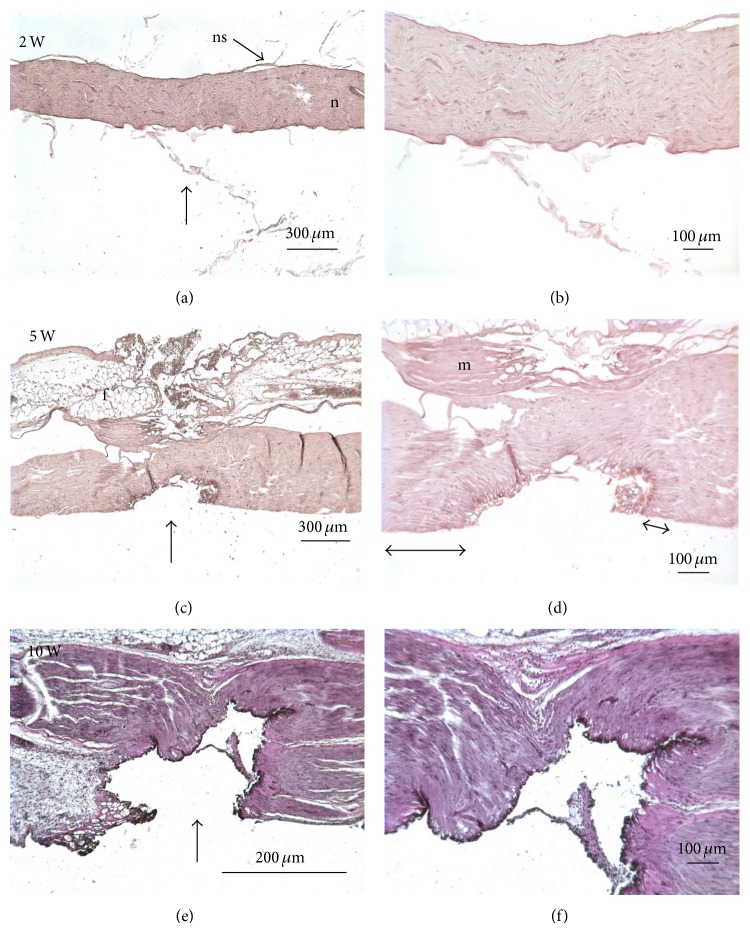
Hematoxylin stained nerves (vertical arrows) exposed to different laser powers: 2, 5, and 10 Watts (W). The left column shows sections at low magnification; the right column shows the same samples at a higher magnification. At 2 W laser power setting, the neural sheath of the nerve was ablated without obvious additional damage to the nerve bundle ((a) and (b)). At 5 W and 10 W laser power settings, severe ablation of the nerve is evident ((c), (d), (e), and (f)). Also note the margin of deeper thermal damage evidenced by staining differences (double headed arrows).

Figures 5 and 7 show representative examples for nerves exposed to 2, 5, and 10 W laser power setting.  Figure 5(a) illustrates the nerve bundle (n) and the nerve sheath (ns), and Figures 5(c) and 5(d) show surrounding fat (f) and muscle tissue (m). After the nerve was exposed to pulses at a laser power setting of 2 W (corresponding to 90 ± 6 mJ/pulse) surface tissue ablation with minor nerve sheath damage, but no nerve bundle changes could be observed by histology (Figures 5(a), 5(b), and 7(a)). At 5 W laser power setting, which corresponds to 230 ± 20 mJ/pulse, the nerve sheath and bundle have been locally ablated (Figures 5(c), 5(d), and 7(b)). Subtle changes in staining bordering the ablation area are evident (Figure 5(d), double arrows) and are typical of thermal damage from laser energy. Increasing the laser power setting to 10 W, which corresponds to 480 ± 30 mJ/pulse, leads to severe ablation of the nerve (Figures 5(e), 5(f) and 7(c)).

**Figure 6:**
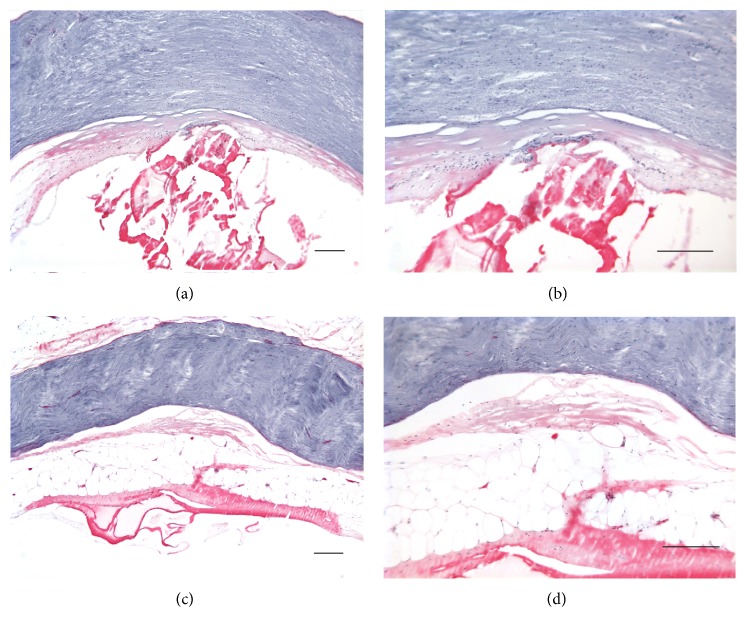
Luna stain of nerve sections is shown for 30 MALIS parallel to the nerve ((a), (b)) and 40 MALIS perpendicular to the nerve ((c), (d)). Forceps tips were 0.5 mm deep and were touching the nerve. The left column shows sections at low magnification; the right column shows the same samples at a higher magnification. Tissue damage is indicated by the red Luna stain but does not visibly extend to the nerve itself. Calibration bars are 200 *μ*m.

**Figure 7:**
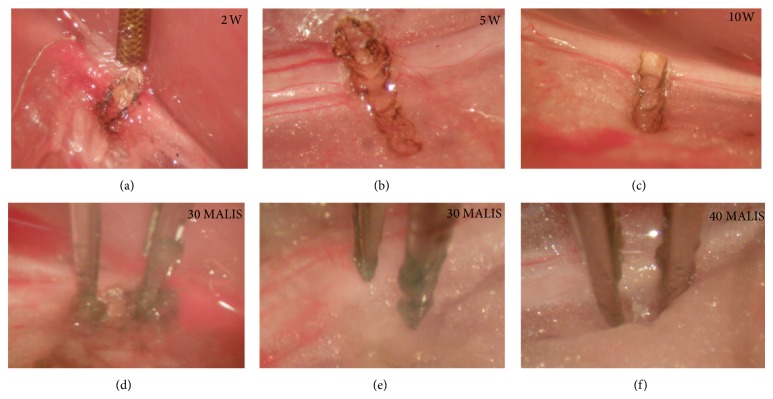
Sciatic nerve images following use of the laser ((a), (b), and (c)) and the MALIS ((d), (e), and (f)) to ablate tissue. The laser created a carbonized track (dark edges) traversing the sciatic nerve. Only small carbonization marks were visible after the use of the MALIS.

Figure 6 shows Luna stain of nerve sections for 30 MALIS power setting parallel to the nerve ((a), (b)) and 40 MALIS perpendicular to the nerve ((c), (d)) with tips 0.5 mm into the tissue and touching the nerve. Figures 7(d), 7(e), and 7(f) illustrate the parallel and perpendicular positions of the tips and tissue condition after activation. No damage to the nerve was detectable if the forceps tips were at the surface of the tissue. If the tips of forceps were inserted into the tissue, heat related changes can be seen in tissue surrounding the nerve for 30 or 40 MALIS power settings. Major physical damage could only be achieved by operating the MALIS with the nerve held between the forceps tips.

### 3.2. Nerve Function

#### 3.2.1. Following Use of Laser

**Figure 8:**
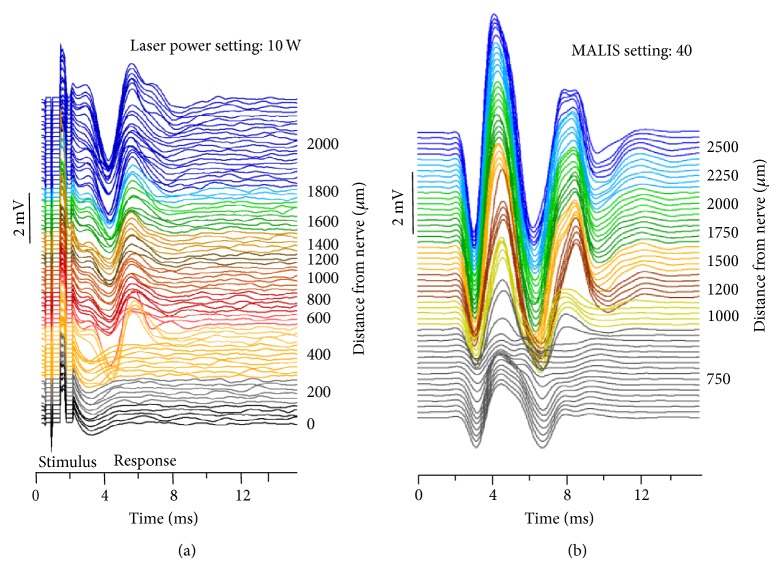
Amplitude (mV) of compound nerve potentials (CNPs) at varying distance (*μ*m) from the nerve. Replicate measures at a given distance have a similar color. (a) shows 10 W laser CNPs and increasing proximity to the nerve severely reduces nerve function. (b) shows that, at 40 MALIS power setting, increasing proximity to the nerve reduces but does not eliminate nerve function.

**Figure 9:**
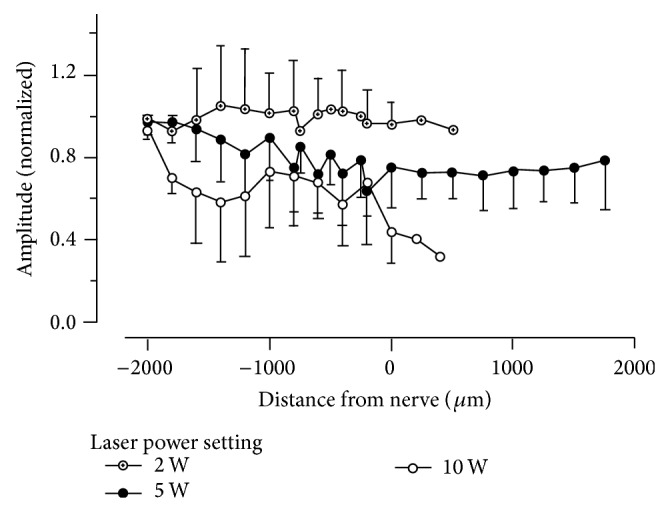
Recordings of the compound nerve potentials (CNPs) amplitudes for different distances from the sciatic nerve. Each data point is an average of at least five replicate CNP amplitudes normalized to a series of recordings made before any laser pulse had been delivered. Because the left and right sciatic nerves were often used for different power settings, an individual animal may be represented in two different power setting data sets. The number of replicates *N* is therefore the number of independent nerves. 2 W (*N* = 5), 5 W (*N* = 9), and 10 W (*N* = 5) from a total of 11 animals. For 2 W, no change in amplitude and hence nerve function can be observed. The results are more variable for 5 W and 10 W. However, the outcomes for the 10 W laser power setting are more deleterious to function than for the 5 W laser setting. Power setting amplitudes are significantly different by ANOVA (*P* = 0.05).

Figure 8(a) shows the changes in nerve responses following use of laser at 10 W power. The *x*-axis is the time from electrical stimulus and the left *y*-axis shows the amplitude of the electrically evoked CNPs. Replicate measures at a given distance (right *y*-axis) of the laser firing lateral to the nerve have a similar color. The CNP peak-to-peak amplitude response decreased gradually with decreasing distance of the fiber from the nerve and very close to the nerve the amplitude of the response was eliminated. To quantify the changes in CNP amplitude with decreasing distance from the nerve, the amplitudes between lasing events were averaged and the values are shown versus the distance of the center of the laser fiber from the nerve (Figure 9). Figure 9 shows that for 2 W laser power setting the nerve function was not affected. Increasing the laser power setting to 5 W leads to more variable results, in that for some nerves the laser did not change nerve function, while for others the CNP amplitude was decreased to less than half of the starting amplitude. For the 10 W power setting nerve function was severely impaired once the beam path crossed the nerve. Amplitudes of each power are significantly different from each other. The plots in Figure 8 also demonstrate that the distance of the fiber from the target structure matters. While the first changes in CNP amplitude can be seen at the 5 W setting for the laser fiber about 1 mm away from the nerve, changes already occur at 10 W laser power setting if the fiber is 1.8 mm away (Figure 9).

#### 3.2.2. Following Use of the Codman MALIS CMC-III Electrosurgical System

**Figure 10:**
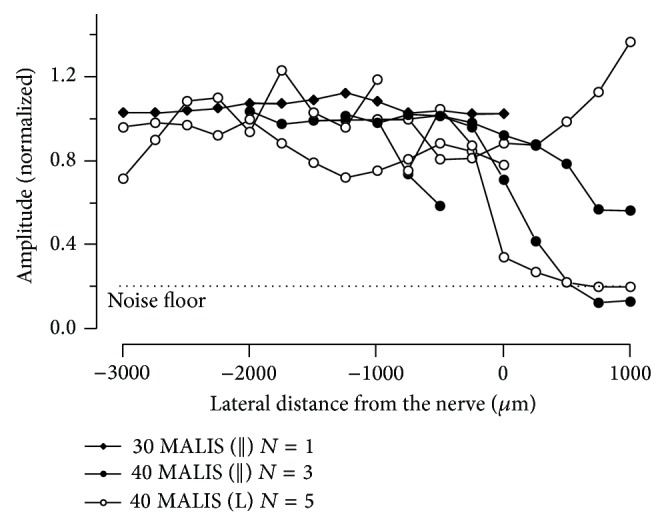
Recordings of the compound nerve potentials (CNPs) amplitudes of nerves immediately after the use of the MALIS at different distances from the sciatic nerve. Each data point is an average of at least five replicate CNP amplitudes normalized to a series of recordings made before MALIS activation. MALIS power settings are indicated as 30 or 40 MALIS with the forceps tip orientation to the nerve in parentheses, that is, parallel (*||*) or perpendicular (L). Because the left and right sciatic nerves were often used for different treatments, an individual animal may be represented in two different data sets. The number of replicates *N* is therefore the number of independent nerves. 30 MALIS (L) *N* = 1, 40 MALIS (*||*) *N* = 3, and 40 MALIS (L) *N* = 5, from a total of six animals. Power setting amplitudes are significantly different by ANOVA (*P* = 0.05).

Operating the MALIS at power setting of 30 MALIS at increasing proximity to the nerve did not change the CNP amplitude. This was true whether the tips were on the surface of the tissue or were pushed about 1 mm deep into the tissue at similar distances from the nerve. Placement of the tips of the forceps onto the tissue, including the nerve itself, did not significantly change the nerve function. Parallel nerve surface placement of the forceps tips was not possible due to the frequent physical displacement of the nerve by the forceps. Inserting the forceps tips into the tissue did not change CNP amplitude either. Even at zero distance from the nerve for the perpendicular configuration, where the nerve lies between the forceps tips, nerve function remained unaffected at 30 MALIS power setting and visual inspection through the dissection scope indicated only some change in tissue structure. In a separate set of measurements, the power setting was selected at 40 MALIS. Again, the cutting mode was used with tips perpendicular to the nerve, and the system was activated for 2 s.  Figure 8(b) shows the corresponding changes in nerve responses over time. The *x*-axis is the time from electrical stimulus and the left *y*-axis shows the amplitude of the evoked CNPs. Replicate measures at a given distance of the MALIS activation lateral to the nerve have a similar color. The CNP peak-to-peak amplitude response decreased gradually with decreasing distance (right *y*-axis) of the forceps tips from the nerve.  Figure 10 shows that 40 MALIS power setting with tips in contact with the nerve in either parallel (*||*) or perpendicular (L) orientation can cause loss in CNP amplitude and hence loss of function. Placing the tips of the forceps perpendicular to the nerve so as to hold the nerve between the tips (0 distance) frequently caused the most tissue ablation and loss of CNP amplitude. Some reduction in nerve function occurred between 1 and 2 mm away from the nerve.

## 4. Discussion

The data presented in this paper compares the functional and physical effects on a nerve due to operation of a bipolar cautery device and a CO_2_ laser, such as what may be encountered when performing a tumor dissection near vital neural structures [19, 22]. Others have shown that coagulation of arterial and venous blood vessels with lumens in the range of 1 mm or larger is more efficient using a bipolar cautery than using lasers [23, 24]. However, the CO_2_ laser is well suited as both a dissecting and coagulating tool when nerve function is taken into consideration because it can be operated contact-free in small and poorly accessible sites [25].

We have provided real time neural function data to model collateral damage effects of microsurgery near the rat sciatic nerve, using a CO_2_ laser or bipolar electrocautery. We demonstrate the advantages and disadvantages of both tools in our model system, with the caveat that a direct comparison between the CO_2_ laser and the MALIS bipolar cautery forceps is complicated because the delivery of energy to tissue differs between them. The MALIS bipolar cautery forceps only causes a tissue interaction where there is physical contact with the tissue allowing passage of an electrical current, while a laser does not require contact. The effect of electrocautery forceps on tissues is dependent on insertion depth and the electrical current field. For example, superficial contact between the forceps tips and the tissue results in a different current path than with deeper insertion. A greater heating and cautery effect occurs with deeper insertion and the charring of proximal tissue adds another variable, which also changes the current.

At the lowest (2 W) laser power setting, functional changes in the rat sciatic nerve were absent and the nerve was essentially undamaged, which is consistent with our histology findings and of others who have described no damage to nerves at 2 W with exposure of swine brain pia membrane to a CO_2_ laser. Also, their histology findings of neural damage at 7 W are similar to our findings of damage at 5 W [26], with the caveat that laser settings and other parameters vary slightly between studies. The 2 W setting of the OmniGuide CO_2_ laser system can therefore be used with reasonable safety close to neural structures in the single pulse mode. Higher power settings of 5 and 10 W are efficient at vaporizing dense fibrous tissue without contact and are good for maintaining visibility of the surgical site but have much greater potential to damage neural structures with a single pulse and should be used cautiously, especially near neural tumor margins. In fact, at the 10 W power setting, the rat sciatic nerve was almost transected, severely damaging nerve function. The thermochromic ink model demonstrates that collateral heating and potential to cause functional damage extend deep into the tissue in the form of an ablation cone.

The Codman MALIS CMC-III Electrosurgical System could be used at the 30 MALIS power setting without damaging the sciatic nerve. We only achieved major damage to the nerve at 40 MALIS power setting with the nerve held between the forceps tips. The MALIS can therefore be used safely to cauterize small blood vessels adjacent to the nerve without damage if the operator assures not to interpose neural tissue. In contrast to the laser, the energy is well localized to tissue placed between the forceps tips; however, the surgical view can become restricted by carbonized tissue, unlike the laser, which vaporizes the tissue. This carbonizing effect and adherence of tissue to the tips has been described by others and we also had to clean the tips despite them being designated “nonstick” [23, 26, 27]. The thermochromic ink model showed that the heat generated is highly localized between the tips. The potential tissue damage is therefore mostly limited to tissue between the tips, with depth of damage determined by how deep the tips are pushed into tissue, unlike the laser. Using the lower power setting would therefore reduce the potential for collateral tissue damage but may require the target structure to be squeezed with the forceps increasing the potential for mechanical damage.

In summary, proximity of the laser beam to the nerve gave transient changes in electrophysiological nerve function measures, while the bipolar forceps gave an all or nothing nerve ablation and damage from the laser beam placed above the nerve was dependent on beam energy. The narrow laser beam path therefore allows close approach to the nerve without contact as previously suggested [28, 29]. In conclusion, surgical techniques that require proximity to nerves benefit from exploiting the combined properties of laser and bipolar forceps.

## 5. Clinical User Recommendations

1. Most importantly, it should be emphasized that only experienced surgeons should use a CO_2_ laser system because in neurosurgical procedures the pathologies are commonly in close proximity to nerve structures. Understanding the precise nature of laser tissue interaction as well as electrical tissue interaction can augment the safety margin of the surgeon employing these tools.
2. The data provides guidelines for appropriate laser power settings for tissue ablation. When the operator is working in close proximity to the neural structures with the option that the neural structure is in the laser beam path, a laser power setting of 2 W or less should be employed. However, in cases of fibrous lesions such as some schwannomas or meningiomas, the use of laser power settings greater than 2 W may be necessary.
3. The MALIS bipolar system can be operated even in contact with the nerve provided it is not allowed to come directly between the forceps tips.
4. We advocate the combined surgical approach utilizing CO_2_ laser in combination with MALIS bipolar system, especially when operating on firm fibrous lesions as long as the operator understands and keeps the power settings and technique in line with the recommendations and observations outlined in this study.
